# Syndemic lives of women who smoke and experience homelessness: secondary analysis of US qualitative data

**DOI:** 10.1093/heapro/daaf216

**Published:** 2025-12-23

**Authors:** Kate Frazer, Maya Vijayaraghavan

**Affiliations:** Health Sciences Centre, University College Dublin, Belfield Campus, Dublin 4, Ireland; Fulbright Scholar, Center for Tobacco Control Research and Education, University of California, San Francisco, CA 94143-1390, United States; Division of General Internal Medicine, University of California, San Francisco, CA 94143, United States

**Keywords:** tobacco cessation, people experiencing homelessness, social determinants of health, social ecological model, syndemic

## Abstract

Tobacco use remains the leading cause of preventable deaths worldwide. Despite efforts by many countries to reduce the impact of tobacco products and lower smoking rates, smoking prevalence is higher among people experiencing homelessness. The reasons are complex and go beyond individual choices, including limited awareness about quitting, restricted access to services, and previous negative experiences. This study is a secondary data analysis of qualitative interviews with women experiencing homelessness. The original data were collected in 2021 as part of a study aimed at understanding tobacco behaviours, attitudes towards quitting, and factors influencing engagement for a subsequent randomized controlled trial. This secondary qualitative analysis, conducted in 2025, examines how policy and environmental contexts influence smoking behaviours among women experiencing homelessness using a social ecological model (SEM) framework. Data were categorized and mapped using the five specified categories within the SEM: policy and environment, community contexts, organizations and systems, interpersonal connections, and individual power and resource distribution. The participants show remarkable resilience in overcoming early-life and adolescent environmental and contextual hardships. Gaps in health services emphasize the need for aligned policy and service improvements. Using the SEM framework, which emphasizes the policy and environmental contexts, offers a deeper understanding of the context of women’s lives. The evidence from this study supports the implementation of gender- and age-appropriate holistic approaches to healthcare and tobacco interventions tailored to this community.

Contribution to Health Promotion statement

- Building supportive health-promoting environments is a fundamental pillar of health promotion, and current evidence presents compounded health and social syndemic challenges faced by people experiencing homelessness.
- Environmental health is critical to personal health and wellbeing.
- Women who experience homelessness require supportive policies and environments embedding a strengths-based approach using co-designed and holistic health programmes, including for tobacco control.

## INTRODUCTION

Homelessness is a complex issue influenced by multiple social determinants of health (SDoH)—a wicked problem (Rittel and Webber 1973). The SDoH have a profound impact on health outcomes and contribute to disparities (Marmot 2005, 2020, Marmot and Allen 2025, WHO 2025). People experiencing homelessness lack access to healthcare, including preventive care, and often present late for treatment, thus living poorer lives with untreated chronic health conditions in unsafe environments (Baggett *et al*. 2010, Fazel *et al*. 2014). Additionally, those experiencing homelessness are more likely to have smoking rates four to six times higher than the general population (Baggett *et al*. 2013, Vijayaraghavan *et al*. 2020). Contrary to popular belief, they have been interested in quitting for more than a decade, as we learned that around 40% made a quit attempt in the past year (Baggett *et al*. 2013, Vijayaraghavan *et al*. 2020). However, they are less likely to be invited to attend services, and persistent barriers hinder their access to traditional smoking cessation programmes (Vijayaraghavan *et al*. 2020). This presents an even greater challenge for women experiencing homelessness due to intersectional issues alongside limited access to healthcare despite smoking behaviours and previous quit attempts (Riley *et al*. 2022).

## WOMEN EXPERIENCING HOMELESSNESS

Women experiencing homelessness face specific challenges and vulnerabilities due to gender-related factors, including being single parents, domestic violence, sexual abuse, and a lack of safe spaces (Bretherton and Mayock 2021). They often have limited access to healthcare, such as prenatal and gender-specific services (Milaney *et al*. 2020, Radcliffe *et al*. 2024). Radcliffe notes that, while homelessness is a context-dependent concept that is hard to define, many measurement methods used in healthcare and research often exclude women intentionally (Radcliffe *et al*. 2024, p. 1). Similarly, evidence from the US shows that ‘no contemporary examination of rates of female homelessness in various contexts and with various comparison groups has been conducted’ (Tsai and Lampros 2025 , p. 103), despite women making up about 43% to 45% of those experiencing sheltered homelessness from 2018 to 2022 (p. 104). O’Rourke *et al*. (2024) suggest that biological sex and gender identity are significant factors affecting vulnerability to ill health in syndemics (p. 1). Thus, structural racism, health inequalities faced by women (WHO 2025), as well as policy and economic factors, all contribute to their syndemic conditions (Mendenhall 2017, p. 889, Mendenhall and Singer 2020; Pirrone *et al.* 2021).

## SOCIAL ECOLOGICAL MODELS

Social ecological models (SEM) can explain how SDoH influence health outcomes (Bronfenbrenner 1995, 1999). Golden *et al*. (2015) specific SEM framework focuses specifically on how health-related policies and environments at the centre impact upstream interventions within five interconnected contexts.

- **Health-related policy and environments:** Focus on policies promoting healthy, autonomous decisions, including laws, ordinances, minimum wage, taxes, smoking bans, parks, communication, and transportation.
- **Community contexts in which decisions about policy and environmental change are made**: Immediate infrastructure recognizes local communities’ roles in health disparities, advocating for policy and environmental solutions through decision-making bodies like councils and hospitals.
- **Organizations that monitor and promote policy and environmental change**: Organized groups of people around an issue—advocacy groups. Resourced, connected organizations that monitor and promote policies and environments: examining groups that support policy and environmental change.
- **Interpersonal connections that foster collective action**: Informal social networks or groups formed to support advocacy, connections that foster policy and environmental actions. Examining social networks, trust and facilitating change.
- **Distribution of resources and power across individuals:** People's ability to live and meet daily needs, control their lives, fair resource distribution, and empowering disadvantaged populations in policy-making.

This brief report presents a secondary qualitative analysis of data to gain a deeper understanding of women’s lives and their smoking histories, mapping data to the SEM and examining interventions, policy, and contexts. Using secondary qualitative data analysis methods enables us to gain insights that were not presented in primary analysis (Hughes and Tarrant 2020, Cheong *et al*. 2023).

## METHODS

### Study design

This is a secondary qualitative analysis of primary data previously reported (Miller *et al*. 2023). The analysis was completed from April to August 2025. The reasons for accessing original transcripts were the following: (i) pragmatic due to time constraints; (ii) ethical, to avoid retraumatising women engaging in a once-off interview without a subsequent intervention; and (iii) the data would be reanalysed sensitively to understand their lives. Using primary data for secondary analysis helps reinterrogate limited literature and reduces the impact of repeated interviews with marginalized populations (Hughes and Tarrant 2020, Cheong *et al*. 2023).

### Secondary analysis research question

The question underpinning this secondary analysis was how does the policy and environmental context of women who are experiencing homelessness influence their smoking behaviours?

### Setting and participants

The original study was conducted in San Francisco, California, and recruitment took place at a Safety Net clinic providing clinical care to individuals experiencing homelessness (Miller *et al*. 2023, 2025). Primary data were collected between April and July 2021 (during the COVID-19 pandemic). Eligibility screening was used to select participants, and a brief questionnaire was administered to collect demographic, racial, ethnic, and tobacco use data (Miller *et al*. 2023).

The inclusion criteria were the following:

- Aged 18 years+
- Engaged in primary care and had a primary care provider at the Safety Net clinic in San Francisco
- Currently smoking cigarettes (daily smoking defined as smoking 100 cigs in lifetime, smoking daily in the past 7 days, and those who report smoking daily at the time of the survey. Those who met the inclusion criteria and reported not smoking daily at the time of the survey were classified as non-daily smokers)
- Housing status determined by electronic health record data and confirmed during eligibility assessment
- Homelessness is defined by the federal definition of homelessness in the US (e.g. an individual or family who lacks a ‘fixed, regular, and adequate night-time residence’, including people living in emergency shelters).

Data measures quantitative and qualitative

- The original study included several quantitative data variables, including age, race, ethnicity and tobacco use characteristics. We report descriptive statistics.
- In-depth interviews lasting 30 to 60 minutes were completed with 10 women following consent. Interviews were recorded, with permission, and transcribed verbatim.

### Ethical approval

Original ethical approval was provided by the University of California, San Francisco (UCSF) Institutional Review Board (IRB) (#IRB 20-31627). For this secondary analysis, additional ethical approval was sought from the IRB in April 2025 to access an anonymous dataset and transcripts, and was approved on 29th April 2025, IRB #: 20-31627, Reference #: 438089.

### Positionality

The study team and secondary analysis authors have extensive experience working with homeless and marginalized communities. The current authors are healthcare professionals and females with expertise in building trust with marginalized communities in USA and Ireland. KF, a Fulbright Scholar from Ireland, gained valuable real-world experience volunteering at the Project Homelessness Connect Centre in San Francisco, engaging with people experiencing homelessness, healthcare workers, and academics.

### Data analysis

A qualitative descriptive approach was employed for the secondary data analyses (Doyle *et al*. 2020 ). The lead author (K.F.) read 10 transcripts, and the data were deductively coded using the *a priori* SEM template (Golden *et al*. 2015) categories, which were reviewed by M.V. Descriptive statistics are reported for demographic and tobacco control variables.

## RESULTS

Participants (*n* = 10) had a mean age of 51.44 years (SD = 9.92): eight smoked daily, two non-daily. Eight wanted to quit; seven reported a healthcare professional recommended quitting in the last year (Table 1).

**Table 1.** Demographics and smoking characteristics.

Variable	Percentage (*N* = 10)
Gender	
**Female**	100% (*n* = 10)
Ethnicity	
**Hispanic/Latino**	20% (*n* = 2)
**Not Hispanic/Latino**	80% (*n* = 8)
Race	
**American Indian/Alaska Native**	10% (*n* = 1)
**Black/African American**	30% (*n* = 3)
**White**	40% (*n* = 4)
**Other**	20% (*n* = 2)
Education	
**Less than high school**	20% (*n* = 2)
**High school**	40% (*n* = 4)
**Some college**	30% (*n* = 3_
**College or professional training**	10% (*n* = 1)
Age	
**Mean (SD) Median (IQR)**	51.44 (9.92); 51 (19.5) Range 41–65
Tobacco and nicotine use	
**Daily use**	80% (*n* = 8)
**Non-daily use**	20% (*n* = 2)
Usually smoke menthols or non-menthols	
**Menthols**	50% (*n* = 5)
**Non menthols**	50% (*n* = 5_
Time for first cigarette on waking	
**Within the first 5 mins**	20% (*n* = 2)
**6–30 mins**	30% (*n* = 3)_
**31–60 mins**	10% (*n* = 1)
**After 60 mins**	40% (*n* = 4)
Frequency of smoking a cigarette in a place where you were not supposed to	
**Not at all**	40% (*n* = 4)
**Rarely**	30% (*n* = 3)
**Sometimes**	10% (*n* = 1)
**Often**	20% (*n* = 2)
Intention to quit smoking	
**Never expect to quit**	20% (*n* = 2)
**May quit**	70% (*n* = 7)
**Will quit in the next 6 months**	10% (*n* = 1)
Within the past 12 months, has a doctor or other healthcare provider recommended that you stop smoking	
**No**	30% (*n* = 3)
**Yes**	70% (*n* = 7)
During the past 12 months, have you stopped smoking for 1 day or longer because you were trying to stop smoking	
**No**	60% (*n* = 6)
**Yes**	40% (*n* = 4)

### Secondary analyses

Qualitative data were mapped from original transcripts and categorized under the SEM headings, adopting deductive coding logic. Following the initial categorization of data, the SEM was reviewed and subdivided into facilitators and challenges. Examples of data are reported in Table 2, and a summary is presented below.

**Table 2.** Data mapping to the social ecological model.

	Facilitators	Challenges
Health-related policies and environments	Smoking—seems like it's like dirty and non-socially acceptable, especially, you know, I don't hang out in bars anymore, you can't even smoke, you know, near a bar [laughter] really without it being a burden on, to somebody near. I hate letting my smoke, you know, that I'm smoking, get near somebody or get in the way of their breathing or around them, it makes me feel awful … And my own, I don't like to smoke indoors, I hate, hate any smoke around me. I don't like it when people smoke actually [Cost of cigs is too high]. So anyways, I wouldn't have money back then to buy a pack, let alone, didn't have a cigarette, and so I'd ask somebody for one. They gladly give me one because a pack of cigarillos is only $2.75, they just hand them out like it doesn't even matter. That's the only time I ever smoked those, they're awful. (Participant 1, 41-year-old White woman).	Domestic violence occurred and some guy beat me up, so the guy went on the phone and he said, ‘There's a homeless woman.’ Okay, it took them an hour and a half, almost two hours to show up after the second call. Now I guarantee you if, if he never put that word ‘homeless’ in it would never have taken that long (Participant 3, 56-year-old woman with other race).
	Cut down from 3 packs per day to five cigarettes. Why? Growing up probably, just being more considerate of people around, my surroundings (at home outside) smoking. (Participant 2, 63-year-old White woman).	Came back to look after mother who was unwell. I tried to keep her apartment but as soon as the, as soon as the lease got put in my name my landlord raised the rent from $900 to $2600 within 3 months. And I was working two jobs and I just couldn't afford to keep it so I ended up out on the street, which was really traumatic for me because I didn't have any friends or family here and, you know, I found myself one day standing on a sidewalk with all my mom's antiques and no way to get them anywhere. And I was on the street for a year and half after that. (Participant 4, 51-year-old White woman).
Community Contexts that support and champion policies and environments	I had my first episode of homelessness when I left home to get married. I married a convict in San Quentin. So I left home. Oh, yeah, the, I did a study where they gave me, over the phone, they mailed me $25 and I did the patches. Never the gum, just the patches You know, they had said because of my kidney and my liver, right, about smoking, I have high blood pressure and I have, I'm a diabetic number two somewhat and high cholesterol and allergies and stuff like that. And so she has said, no, the first, Dr. B—— said to me that I should stop and I, I tried to, but then when she told me that I was a diabetic I was about to cry, she just held me like this and I, I know, I just like felt, you know, maybe I should at least try this. (Participant 5, 61-year-old Black/African American woman).	[never offered help to quit] No, but I have high blood pressure now, and so I think that has to do with, maybe, doesn't that have to do with it. (Participant 1, 41-year-old White woman).
	I should quit smoking, though, because my mom has lung cancer, you know, she's in remission but she had lung cancer and I was told years ago. She's doing great, though, and, and, you know,[…] But her doctor about 5 years ago did mention that I should quit. (Participant 1, 41-year-old White woman).	Never offered any quit/cessation supports. (Participant 7, 36-year-old White woman).
	They give me patches and everything. (Participant 6, 65-year-old Black/African woman).	[health care providers recommending quit Q] Uh-uh [no], and they all know I smoke, so, yeah. (Participant 2, 63-year-old White woman).
Organisations That Monitor and Promote policies and environments	I think that would really help people [smoking cessation study] to not only be happy and want to care more about their health, but it distracts them from wanting to smoke and, and it makes you just like more healthy minded. And so that's what I do a lot of times when I'm stressed out or what, what have you, or if I like really want to smoke a cigarette and I'm some, like indoors or in my hotel, I can just start painting and I don't even think about smoking anymore. (Participant 1, 41-year-old White woman).	So it’s a them and us, and so it has a stigma And I went to see my doctor and she, she told me to call next time because this is a waste of time, she needs to be at the hospital ‘and I was just like—well it hadn’t been there for a while and it's like I'd, why is my circumstances a waste of time?’ She doesn't believe that I have diabetes. She’s never tested me and I, I have my medical records that show it, but…. (Participant 3, 56-year-old woman with other race).
	[Smoking cessation study] I think really good because sometimes it does take someone else to like give you the, to motivate you to do something. (Participant 8, 45-year-old Hispanic/Latine woman).	They're very uncool at [Name of accommodation], they treated us with no respect and it was very hard to, to make the curfew at 10 p.m. because my mom has cancer and dementia and my sisters have kids, and I tried and tried, and sometimes I was a few minutes late and they would act like they're going to kick me out all the time. And then finally they did, right before I got housed. (Participant 1, 41-year-old White woman).
	Then, I graduated, did the program, drug program, got my own house in [name of location], and I stayed there for like a year and then I relapsed…. (Participant 9, 44-year-old Black/African American woman).	
Interpersonal Connections That Foster Collective Action	Yeah, I was clean for twenty years from all drugs and I relapsed, and eventually the drugs just got more important than rent and everything, and I’ve been inside now for 11 years, so, or 10 years in this house, but I was homeless for a while. (Participant 2, 63-year-old White Woman).	Age 11 started smoking …. This is raw, my grandfather, my step-grandfather, he was molesting me so he, I could never speak up about it at the time anyway, but he would give me cartons of cigarettes because he had a [xx] route and went to different stores. (Participant 3, 56-year-old woman with other race).
	I wanted to just keep smoking. [laughter] You know, I didn't feel that it was really necessary for me to quit or even that harmful to me. I was maybe in denial, I'm not sure, but when I found out my mom had lung cancer, you know, that definitely made me, you know, more aware of the, you know, harshness of, you know, [laughter] the effects of tobacco and whatnot. But she smoked a lot, you know, a lot, so I always felt like, well, she got it, she got it but I'm not going to smoke that much. (Participant 1, 41-year-old White woman).	Sexual abuse experienced in home. Well, my mother was murdered and so I lived with my blood, my mother's oldest brother and his wife, and she used to really treat me real bad, you know, when he wasn't there. And so, and my aunt, she was like, when I was like moaning and groaning I’m asleep, you know, I don't know what's going on, [laughter] and so she thought I knew so that's why she treated me so bad at home. (Participant 5, 61-year-old Black/African American woman).
		Started smoking age 19 to cover smell of weed. Uncle smoked in home. ‘Yes, there was cigarettes and cigars’. Oh, I was stressed out, I would stay at home, I just cried and cried and cried because I felt, you know, yeah… I would get high and I would smoke cigarettes… I smoked cigarettes, I smoked cigarettes, you know, in my work, I'd just have a cigarette. (Participant 5, 61-year-old Black/African American woman).
		I got stabbed and I’d get beat up by several different people. They would just pick on me because they thought I was a weak person ….. At age 16, I started smoking. My brother got shot in [Name of place] by [Name added]. My mom used to smoke crack. (Participant 10, 38-year-old Hispanic/Latine woman).
Distributions of resources and power across individuals	It is what it is, what it is really, I wouldn’t be the woman I am today if it wasn’t for those things, you know….. I feel terrible now I showed my children that, ‘Oh, I’m going, I’m, this is my time, I’m going to go relax,’ and what I’d do, I’d go outside and I’m smoking a cigarette or smoking a joint, and it's like…This is my time, mommy time.’ And it’s like, so what does that put into their heads (Participant 3, 56-year-old woman with other race).	Roughly as a child, I was like 10–11 or so, I didn't start until I was at least maybe 16–17 years old, and I was watching my mom one day and I just decided to try it myself.. I was the only child living in my parents’ house.[why I started smoking at 16] It was like everybody always picked on me, I always got into fights as a child. That's the reason I'm all bad, bad-tempered around now, because of being bullied in school. (Participant 7, 36-year-old White woman).
	I didn't start smoking on a regular basis until I was about 16 [smoking cigarettes]. I think they just made me feel older, more mature maybe. I smoked a lot more when I was drinking but I haven't drank in eight years [personal agency to quit now]. And I have a really nice place, it is, I have, I have one of the biggest rooms in the building and my own bathroom and a long hallway and I, I have, it is basically a studio (Participant 4, 51-year-old White woman).	Started as child. Back then, it was just I guess, I thought it was cool, it was like I was being cool at the time because I was young. Now it, it is like every time, I'm stressed or irritated or something goes wrong my nerves start messing with me, it is like I, I automatically just want to smoke cigarettes. (Participant 8, 45-year-old Hispanic/Latine woman).
	When I was homeless on the street, yes, I smoked a lot more. Now I, it is about the same as when I was homeless on the street (Participant 8, 45-year-old Hispanic/Latine woman).	No, I chose to be homeless in Mississippi because of the way my parents were acting, always arguing with me to get a job and everything. I have HIV. I have scoliosis, a curvature of the spine. Disability (Participant 7, 36-year-old White woman).

### Health-related policies and environments

Participants demonstrate effective tobacco control measures, including legislative changes to reduce exposure to smoking, such as policies that restrict the sales of menthol and flavoured tobacco, and raise the age for purchase. Policies on smoking cessation therapies and accessible environments are recognized as supportive (Table 2). Challenges include the lack of supportive policies and environments when experiencing domestic violence, seeking affordable housing, and managing rental expenses.

### Community contexts that support and champion policies and environments

Participants describe that organizations’ interpersonal skills are vital in building connections that foster positive environments, especially among healthcare providers discussing smoking cessation and its effects on health. Participants reported a lack of quit support despite consulting healthcare services for other health issues (Table 2).

### Organizations that monitor and promote policies and environments

The role of smoking cessation facilitators and research programmes, when available, was emphasized by participants in addition to the importance of these broader tobacco control policies reaching marginalized and homeless populations. Positive impacts are outlined as helpful for monitoring, motivating, and distracting oneself. Challenges highlight the environments faced by participants engaging with healthcare providers and providers of homelessness accommodation. The exemplars in Table 2 demonstrate stigma and negative consequences.

### Interpersonal connections that foster collective action, policies, and environments

The role of family and significant others facilitate understanding the consequences of smoking, the link, and the effects associated with lung cancer. Interpersonal connections held challenges as participants recalled family and friends who introduced them to smoking behaviours and described environments where smoking was normalized. Participants describe childhood experiences of sexual abuse and incest and describe dysfunctional and hostile childhood environments (Table 2).

### Distribution of resources and power across individuals

Participants demonstrated how power and control are distributed in their lives as they described their decision-making and noted their quit behaviours, especially when they had space and favourable circumstances. The evidence shows the internal and external factors that drive positive behaviours and healthy environments for their families. The adverse effects of childhood abuse, domestic violence, and traumatic bereavement demonstrate challenging experiences. All participants shared that they grew up in environments where smoking was a common behaviour. Participants describe limited support for emotional regulation, and their actions in late adolescence and early adulthood involved efforts to escape from home life, including choosing homelessness and histories of smoking behaviours (Table 2).

## DISCUSSION

Participants in the current study recalled histories of smoking behaviours from childhood, when tobacco control legislation was minimal. They noted that, as adults, they adjusted their behaviours in response to tobacco control measures. Tobacco control legislative changes, including shifts in social norms and bans on smoking in public places, produced positive outcomes in California [CDPH 2020] and globally (WHO 2005). Referral and access to mainstream healthcare services are described as challenging; however, the research study, along with the positive aspects of a proposed smoking cessation programme and the value of one-to-one conversations, were motivating factors (Miller *et al*. 2023). Direct foregrounding of policy and environmental factors highlights the impact of the SDoH (WHO 2003, 2025, Golden *et al*. 2015, Miller *et al*. 2023).

Participants faced increased difficulties during the COVID-19 pandemic, consistent with challenges reported by marginalized communities worldwide (Bambra *et al*. 2020, Connolly *et al*. 2023). They describe their social norms from childhood, multi-generational trauma, their networks, and traumatic environments, all of which had long-lasting effects on their early-life choices and influenced later behaviours. It is unclear whether any women in this study received support or counselling as children or adults, as this information was not gathered and was beyond the scope of the original study. The descriptions of homelessness depict unsafe environments with episodes of negative interactions, including histories of child abuse, neglect and domestic violence; living unsheltered on streets, in cars, or with others in insecure settings, as well as lacking access to healthcare, alongside the absence of supports and assistance. Their experiences are consistent with women who experience intimate partner violence and endure prolonged episodes of homelessness and insufficient support (Hargrave *et al*. 2024). Safe and secure housing policies and environments are essential for wellbeing (Wenzel *et al*. 2001, Posada-Abadía *et al*. 2021, Frazer *et al*. 2025).

Baum *et al*. (2024) remind us of the everyday conditions necessary for creating health equity from early childhood—the first 1000 days. This includes ‘ensuring access to adequate housing’ (p. 25), ‘supportive environments’, and ‘building health systems based on comprehensive primary health care’ that are sensitive to the needs of all people, including ‘women’ (p. 25). The evidence from these 10 women supports this stance.

Death and grief were described through events from childhood and early adulthood. The tragic and violent deaths of family members and partners are documented without evidence of supportive environments to help cope with bereavement and loss. Pathways into homelessness in California reveal economic and broader structural barriers, as well as highlighting deficiencies supporting mental and behavioural health (Assaf *et al*. 2025) and offer opportunities to incorporate health and tobacco treatments into non-clinical settings (Hawes *et al*. 2025). In the UK, Cox *et al*. (2022) also report systems issues for people experiencing homelessness with incomplete screening and only 12% linked to cessation services. Rubin *et al*. (2021) highlight dissonance in current US smoking cessation programmes for women who experience homelessness due to their unmet needs, and call for systemic change. Oestrogen, menopause, and impulsivity are linked to women's tobacco use (Riley *et al*. 2022, p. 6); and Antin *et al*. suggest the global rise in women's smoking is a symbol of emancipation that has been further exploited by tobacco companies using targeted marketing (2017, p. 2). Gaps exist in bereavement and psychological support and for women’s health across the life course. Support, including tobacco treatment, must be tailored to consider the challenging environment.

This analysis highlights women who overcame challenging personal and structural circumstances, or ‘syndemic phenomena,’ yet participated in a research study supporting positive health and tobacco control. Their willingness to participate demonstrates intrinsic motivation, which can be seen as an affirmation of their desire to quit—a strengths-based approach. Economic benefits were not the primary motivator for many; instead, they emphasized the wider impact of quitting, giving back altruistically, and protecting their families.

Challenges for researchers interviewing people who experience homelessness for tobacco control studies have reported gaps in training, although progress has been made in supporting researchers’ wellbeing (Alway *et al*. 2024). The use of trauma-informed training is a valuable tool for tobacco control researchers engaging with people experiencing homelessness, as it demonstrates standard solutions and positive policy interventions that can be implemented quickly to improve health and wellbeing (Alway *et al*. 2024). However, the same level of support does not seem to exist for women in this study or in previous research (Rubin *et al*. 2021).

## LIMITATIONS

This is a secondary analysis of data transcripts from an original study, and participants may be influenced by local and state tobacco control policies and homeless services (Miller *et al*. 2023, p. 7). Qualitative findings are not generalizable by design, and although this analysis is based on 10 accounts, these experiences might be similar for others or may underrepresent their syndemic conditions. The analyses are constrained from data reported in original transcripts; however, it is a method that enables reinterrogation of existing datasets (Hughes and Tarrant 2020). The targeting of women by tobacco industry marketing strategies throughout their lives should be considered (Antin *et al*. 2017).

## CONCLUSION

A SEM framework presents intersectional lives and contexts. This study supports gender- and age-appropriate holistic tobacco and health interventions and policies, considering women’s lives and ability to remain engaged with systems despite the structural barriers and challenges encountered.

## Data Availability

The data in this study are confidential, in keeping with the study's original ethical approval and design.
